# A Mobile Virtual Reality–Based Intervention for Stress Reduction Among Young Adults: Pilot Randomized Controlled Trial

**DOI:** 10.2196/83438

**Published:** 2026-08-21

**Authors:** Houlin Hong, Matthew R Lootens, Rebecca Bachman, Luis David Olivera Leon, Ann F Dunlap, Emilio Goldenhersch, Nicolas Rosencovich, Cristian Waitman, Katarzyna E Wyka, Terry T-K Huang

**Affiliations:** 1 Center for Systems and Community Design City University of New York, Graduate School of Public Health and Health Policy New York City, NY United States; 2 NYU-CUNY Prevention Research Center New York City, NY United States; 3 MindCo Health San Francisco, CA United States; 4 Universidad de Flores Laboratorio de Investigación en Neurociencia y Ciencias Sociales Ciudad Autónoma de Buenos Aires Argentina; 5 Universidad Nacional de Córdoba Escuela de Ingeniería Biomédica Córdoba Argentina; 6 Universidad Siglo XXI Córdoba Argentina

**Keywords:** virtual reality, stress management, young adults, mindfulness, randomized control trial

## Abstract

**Background:**

Young adults experience disproportionately high levels of stress, creating significant health challenges. This demographic often underuses traditional stress management methods due to perceived ineffectiveness, inconvenience, or stigma. Given these challenges, mobile virtual reality (VR) offers a novel, engaging, and accessible alternative stress management tool that may be more appealing to young adults.

**Objective:**

This pilot randomized controlled trial (RCT) evaluated the preliminary effects of MindCo Relief (MR), a mobile VR-based stress management app, accompanied by online coaching, on perceived stress, resilience, anxiety, depressive symptoms, and quality of life (QoL).

**Methods:**

Young adults aged 18 to 29 years were recruited to participate in the study. A total of 70 participants with moderate-to-high stress levels at baseline were randomized to an intervention (n=34) group and a control (n=36) group. Moderate-to-high stress was defined as a sum score of 14 or more on the Perceived Stress Scale–10 (PSS-10). The intervention group completed an 8-week VR mindfulness program with access to in-person coaching, while the control group received no intervention. The primary outcome was self-reported perceived stress (PSS-10). Secondary measures included resilience (measured by the Connor-Davidson Resilience Scale–2), anxiety (measured by the Generalized Anxiety Disorder–7 scale), depressive symptoms (measured by the Patient Health Questionnaire–9), and QoL (measured by the General Well-Being Schedule). Primary analyses used linear mixed-effects models to assess the intervention effect over the 8-week study period. Secondary analyses examined whether the percentage of program modules completed and the number of coaching sessions attended were associated with changes in the primary and secondary outcomes.

**Results:**

The mean age of the participants was 23 (SD 3.41) years, and they were predominantly female (n=52, 74%), students (n=46, 66%), and insured (n=63, 90%). No significant differences were observed between the intervention and control groups in terms of sex (*P*=.89), student status (*P*=.56), insurance status (*P*=.68), race and ethnicity (*P*=.18), or age (*P*=.32). Linear mixed-effects models showed a significant interaction between time and intervention, with the intervention group showing greater reductions in stress (β=−3.84, SE 1.37; *P*=.007), anxiety (β=−3.21, SE 0.97; *P*=.002), and depressive symptoms (β=−3.13, SE 1.13; *P*=.007) and a greater increase in QoL (β=9.99, SE 2.92; *P*=.001). No significant interaction was found for resilience (β=0.83, SE 0.43; *P*=.06). In the secondary analyses, neither the percentage of modules completed nor the number of coaching sessions attended was significantly associated with changes in the primary or secondary outcomes.

**Conclusions:**

This study provided preliminary evidence that mobile VR can help reduce stress among young adults by providing immersive environments that promote relaxation and adaptive stress responses. The findings support further exploration of VR’s role in mental health care. Future research should examine the efficacy and long-term effects of this mobile VR software using larger, more diverse samples. Strategies to sustain VR engagement are also warranted.

**Trial Registration:**

ClinicalTrials.gov NCT06970652; https://clinicaltrials.gov/study/NCT06970652

## Introduction

Young adults aged 18 to 29 years in the United States experience disproportionately high levels of stress, making them one of the most vulnerable groups for mental health disorders [1-3]. Several often co-occurring challenges of young adulthood, such as pressures to succeed in school, establish a career, maintain relationships, and manage finances, contribute to higher stress levels in this age group than among older adults [2,4]. Repeated exposure to social comparisons and expectations from frequent use of social media and other digital technologies may further exacerbate feelings of stress [5,6].

Stress can have a profound impact on both mental and physical health. Studies have shown that prolonged stress is a precursor to severe psychiatric conditions, such as major depressive disorder, bipolar disorder, and posttraumatic stress disorder (PTSD) [7]. Furthermore, adverse psychological health conditions, including stress, anxiety, and depression, are considered risk factors for neurological, metabolic, and cardiovascular diseases (CVDs) [8,9]. The psychological effects of frequent stress and the perception of daily stress may disrupt short-term stress responses and hinder healthy adaptation. Consequently, these disruptions can increase the risk of chronic diseases such as CVD [10].

For young adults, the consequences of untreated stress extend to diminished academic and occupational performance and reduced overall quality of life (QoL), highlighting the urgent need for accessible and effective interventions [11]. Despite studies showing that physical activity is positively associated with mental well-being, only a quarter of US adults meet the National Physical Activity Guidelines [9,12,13]. Although traditional methods such as cognitive behavioral therapy (CBT) and mindfulness-based stress reduction therapy have also been shown to be effective for managing stress and anxiety, the uptake of these treatments among young adults is often hindered by perceptions that such care is ineffective or inconvenient [14-16]. Studies have also shown that stigma is a major barrier to mental health help seeking among college students, as they feel embarrassed and fear being labeled “crazy” by others [17,18]. Young adults also lack knowledge about mental health services and have difficulty identifying symptoms of mental illness. Consequently, they often continue to normalize their elevated distress and prefer self-reliance over professional help [3,18-20]. Moreover, traditional therapeutic approaches often lack personalization and do not resonate or align with young adults’ individual needs and lifestyles [20].

Given these challenges, innovative stress management interventions that are flexible, accessible, and appealing to young adults are needed. Virtual reality (VR) is a novel and promising stress management tool, providing an immersive and engaging experience that can be more appealing than traditional approaches to many individuals [21]. VR technology uses computer modeling to generate interactive 3D visual environments, with additional auditory and haptic feedback also possible [22-24]. VR allows users to experience and interact with the generated environment as if they were physically present, setting it apart from other non-VR apps, websites, and video-based relaxation programs [25]. Compared to imagery and 2D technologies, VR triggers stronger emotional engagement and physiological responses, making it a more efficient medium for immersive experiences [26]. By fostering a strong sense of presence in a calming, controllable environment, VR can support short-term emotion regulation, which in turn may improve subjective well-being and QoL [27]. Mobile VR operating on smartphones can enhance VR’s accessibility and portability, making it a potential solution for addressing stress on a larger scale [20].

Despite the growing interest in VR apps, there is limited research on the effectiveness of VR in stress reduction and management, particularly among young adults. Some studies have shown promising results, indicating that VR is associated with stress reduction [28-30]. However, most of the literature to date on VR therapy focuses on addictions or severe mental health conditions, such as social anxiety disorder, obsessive-compulsive disorder, and PTSD [20,22,31]. Although mobile technology has evolved rapidly over the past 5 years, studies examining VR use for stress remain scarce [27].

This pilot study used MindCo Relief (MR), a smartphone VR app designed for stress reduction, as the intervention in a randomized controlled trial (RCT) to test whether this mobile VR behavior change program would reduce perceived stress among young adults aged 18 to 29 years. We hypothesized that after the 8-week intervention period, use of the MR app would be positively associated with decreases in perceived stress, depressive symptoms, and anxiety and increases in resilience and QoL.

## Methods

### Study Design

This study was an 8-week pilot RCT conducted to examine the feasibility and preliminary impact of MR on stress reduction among young adults. Block randomization by sex was used to ensure a balanced distribution between male and female participants. The random allocation sequence was generated using a computer-based random number generator, with a block size of 2. Participants were assigned sequentially to the intervention or control group according to the generated allocation sequence. Enrollment occurred from October 2023 to January 2024. The first participant began the trial in October 2023, and the last completed the study in May 2024. This study was reported in accordance with the CONSORT (Consolidated Standards of Reporting Trials) checklist, which can be found in Multimedia Appendix 1.

### Participants

Study participants were screened for eligibility using an online REDCap-based survey (Vanderbilt University) [32,33]. Eligibility criteria included being aged 18 to 29 years, having a moderate or high level of perceived stress at baseline, having access to a smartphone running the Android or iOS operating system, and being able to install and run the MR app. Perceived stress was measured using the Perceived Stress Scale–10 (PSS-10) [34,35]. Individuals participating in other stress reduction programs or with photosensitive epilepsy were excluded. Moderate stress was defined as a PSS-10 score of at least 14 but no more than 26, and high stress was defined as a score of 27 or greater. Participants were recruited primarily through online postings (on university websites and social media platforms) directed at individuals residing in New York City. Recruitment materials emphasized stress management and targeted college students and young adults meeting the inclusion criteria. After enrollment, participants were required to visit our study site to complete randomization.

### Intervention Details

Participants in the intervention group received access to the MR smartphone app and a smartphone-compatible VR headset, both developed by MindCo Health [36]. All participants in the intervention group received VR headsets directly from the research team at our study site. The app contained a stress reduction program that comprised a structured sequence of 76 modules designed to be completed over 8 weeks. Each module was designed to be completed in approximately 7 minutes and had to be completed before progressing to the next module. Forty modules used VR. In these modules, participants placed their personal smartphones running the MR app in the VR headset, and the smartphone provided all the audiovisual content. All other modules used smartphones without the VR headset.

The program was grounded in a CBT framework and integrated mindfulness-based stress reduction principles with structured psychoeducation and immersive natural environments [37-39]. VR modules served 2 complementary functions. First, educational VR sessions placed participants in a simulated therapy office environment where a prerecorded clinician delivered CBT-based psychoeducational content focused on stress physiology, cognitive appraisal, maladaptive thought patterns, and coping strategies. This immersive format was designed to enhance attentional engagement and contextual encoding of therapeutic material. Second, mindfulness-based VR sessions transported participants to simulated natural environments (eg, forests and beaches), where guided practices such as breath awareness, body scan, muscle relaxation, and compassion exercises were delivered within biophilic settings to promote attentional stabilization and relaxation.

VR module completion was automatically tracked through the app backend. To initiate immersive playback, participants were required to grant gyroscope and rotation permissions, activating head-tracking mode. VR videos could not be fast-forwarded, and module completion status was recorded only after full playback duration. Progression to subsequent modules required completion of prior modules. Backend logs captured the playback duration and activation of immersive mode. As is typical in remote digital health interventions, direct verification of sustained headset wear or continuous visual attention was not feasible. Self-reflective journaling modules reinforced the integration of psychoeducational and mindfulness content by prompting participants to identify stress triggers, bodily sensations, and cognitive responses.

In addition to the structured program, the app provided access to 30-minute online coaching sessions. Each participant in the intervention group was allowed to schedule up to 3 sessions during the 8-week participation period. Coaches were non–US-licensed psychologists based in Argentina, trained in CBT, and followed a protocol developed to support the MR intervention. The protocol promoted engagement with the MR modules aligned with the participants’ needs at the time of the coaching session. The control group received no active intervention throughout the study period.

### Primary and Secondary Outcomes

The primary study outcome was perceived stress, measured by PSS-10, a widely used, validated, and easy-to-use questionnaire [34,40,41]. The PSS-10 includes 10 questions, each scored from 0 to 4. A sum score is calculated as the total PSS score, with a higher score indicating a higher stress level. Secondary outcomes were also collected via surveys, including resilience, anxiety, depression, and QoL. Resilience was measured using the Connor-Davidson Resilience Scale–2 (CD-RISC2), with a total score ranging from 0 to 8, where higher scores indicate greater resilience [42]. Anxiety symptoms were assessed using the Generalized Anxiety Disorder-7 scale (GAD-7), and depressive symptoms were measured using the Patient Health Questionnaire–9 (PHQ-9). Both instruments evaluated symptom frequency over the past 2 weeks using a 4-point scale with answer options as follows: 0=“Not at all,” 1=“Several days,” 2=“More than half the days,” and 3=“Nearly every day.” Total scores range from 0 to 21 for the GAD-7 and 0 to 27 for the PHQ-9, with higher scores indicating greater symptom severity [43-46]. QoL was measured using the General Well-Being Schedule (GWS), which comprises 18 items assessing psychosocial well-being; higher scores indicate better overall QoL [47-49]. All measures have demonstrated good reliability and validity in adult populations and have been widely used in young adults of the study cohort [41,50-52]. Assessments for all outcomes were conducted at baseline and again at 8 weeks after the conclusion of the intervention.

### Covariates

Demographic variables were collected from the participants at study enrollment, including age, sex (male vs female), race and ethnicity (Asian, Black, other, Hispanic, or White), student status, and health insurance status (public, private, or none). Age and student status were examined to account for potential differences in digital program use, as recent systematic reviews have indicated that these factors significantly influence adherence to digital health interventions [53,54]. Additionally, race and ethnicity were explored to assess potential differences in intervention outcomes. Reports and studies have shown that mental health outcomes and stress exposure differ across racial and ethnic groups in New York City, with structural and environmental inequities contributing to higher psychological distress among Black and Latino adults [55-57]. A previous study has also indicated that insurance status may affect mental health conditions [57].

### Statistical Analysis

Demographics were summarized by treatment group, and chi-square tests were used to examine differences in categorical variables by intervention status. A 2-tailed *t* test was used to compare age between the intervention and control groups. Additional comparisons were conducted between individuals who enrolled in the study and those who were eligible but did not participate, as well as between participants who completed the follow-up assessment and those who were lost to follow-up.

Linear mixed-effects models with repeated measures were used to regress primary and secondary outcomes on time, intervention, and the interaction between time and intervention. The models also included fixed effects for sex, as sex was used for block randomization. With 2 waves of data collection, a compound symmetry covariance structure was used. Other covariates that were balanced between the 2 groups were not included in the model.

In the secondary analysis, we also examined whether program engagement was associated with improvements in outcomes. Changes in scores for the primary and secondary outcomes were first calculated as the difference between follow-up and baseline measurements and then modeled as a function of program engagement. Program engagement was assessed by the percentage of module completion and the number of in-person coaching sessions attended. Both variables were analyzed as continuous and categorical variables, with each specification evaluated in a separate regression model. When analyzed as categorical variables, the percentage of module completion was divided into quartiles, and the number of coaching sessions was dichotomized as <2 sessions vs ≥2 sessions.

The primary analysis was conducted on an intention-to-treat (ITT) basis. No participants with relevant observed data were excluded, and the analysis followed the participants’ assigned group, regardless of the actual treatment or dose received. The secondary engagement analysis, which modeled change scores as a function of the dose received, was restricted to participants with both baseline and follow-up measurements. Statistical analyses were performed using R (version 4.4.1; R Foundation for Statistical Computing) [58,59]. The α level was set at .05.

### Ethical Considerations

The study was approved by the City University of New York Institutional Review Board (2023-0338-PHHP), which determined the study to be of minimal risk. This study was registered at ClinicalTrials.gov (NCT06970652). All participants were provided with detailed information regarding the study’s purpose, procedures, potential risks and benefits, data confidentiality, and their rights as research participants. Informed consent was obtained electronically through REDCap prior to study participation.

Participation was entirely voluntary, and participants were informed that they could withdraw at any time without penalty. To protect privacy and confidentiality, data were deidentified before analysis, and only the research team had access to the final analytic dataset. Personally identifiable information was stored separately from study data on secure, password-protected servers. Participants who completed all 8 weeks of the study and provided postintervention data were compensated with a US $50 electronic gift card.

## Results

### Sample Characteristics

**Figure 1:**
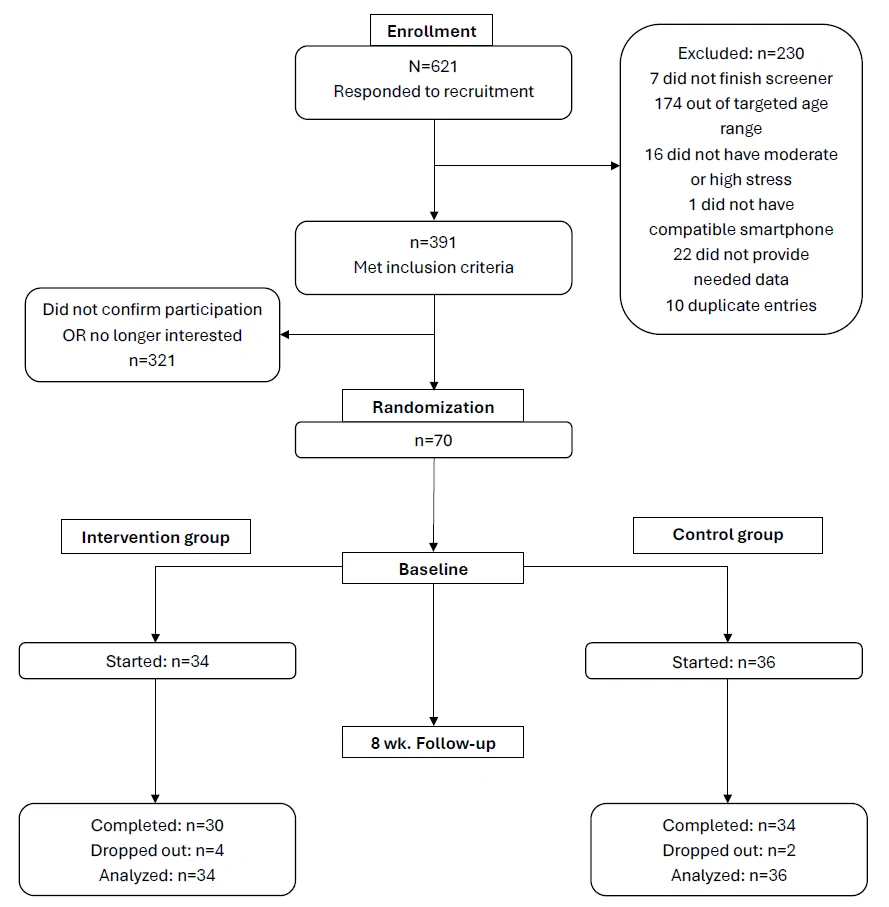
CONSORT flow diagram.

A total of 621 individuals responded to the online recruitment, of whom 230 were excluded based on the study’s inclusion and exclusion criteria. Among the remaining respondents, 283 individuals completed the consent form, and 70 enrolled in the study and were randomized to either the intervention or control group. Figure 1 shows the sampling flowchart. Demographic comparisons between participants who enrolled and those who were eligible but did not enroll are shown in Table S1 in Multimedia Appendix 1.

Of the 70 enrolled participants, 34 were randomized to the intervention group and 36 to the control group. The mean age was 23.4 (SD 3.41) years, and the majority were female (n=52, 74%). Forty-six participants were college students, and 63 reported health insurance coverage, either private (n=24) or public (n=39). We found no significant differences in demographic variables between the intervention and control groups at baseline (all *P*>.05; Table 1). Six participants dropped out of the study and failed to provide postintervention data (n=4 in the intervention group; n=2 in the control group). Additional comparisons showed no demographic differences between those who were lost to follow-up and those who provided follow-up data (Table S2 in Multimedia Appendix 1).

**Table 1:** Demographic characteristics of study participants (n=70).

Characteristics		Overall	Control group (n=36)	Intervention group (n=34)	*P* value	
**Sex, n (%)**						.89
	Male	18 (26)	9 (25)	9 (26)		
	Female	52 (74)	27 (75)	25 (74)		
**Student status, n (%)**						.56
	Not a student	24 (34)	14 (39)	10 (29)		
	Student	46 (66)	22 (61)	24 (71)		
**Insurance status, n (%)**						.68
	Public	39 (56)	22 (61)	17 (50)		
	Private	24 (34)	11 (31)	13 (38)		
	None	7 (10)	3 (8)	4 (12)		
**Race and ethnicity, n (%)**						.18
	Asian	24 (34)	12 (33)	12 (35)		
	Black	9 (13)	4 (11)	5 (15)		
	Hispanic	22 (31)	12 (33)	10 (29)		
	White	11 (16)	8 (22)	3 (9)		
	Other	4 (6)	0 (0)	4 (12)		
Age (years), mean (SD)		23.36 (3.41)	23.75 (3.51)	22.94 (3.30)	.32	

Overall, the sample showed moderate levels of stress (PSS-10: mean 25.87, SD 4.84; 23/70, 33% with high stress), moderate resilience (CD-RISC2: mean 4.52, SD 1.50), moderate anxiety (GAD-7: mean 11.41, SD 4.61), moderate depression (PHQ-9: mean 11.99, SD 6.26), and severe distress (GWS: mean 55.66, SD 13.23). There were no significant differences between the intervention and control groups in perceived stress, anxiety, depressive symptoms, resilience, or QoL at baseline (all *P*>.05).

### Primary Outcome

The intervention group showed a decrease in average stress scores from 25.92 (SE 1.03) at baseline to 18.21 (SE 1.03) after the intervention. Linear mixed-effects regression showed a significant interaction between the intervention group and time for PSS-10 (β=−3.84, SE 1.37; *P*=.007; Table 2).

**Table 2:** Mean scores for primary and secondary outcomes at baseline and follow-up (n=70)^a^.

Outcomes	Control group (n=36), mean (SE)		Intervention group (n=34), mean (SE)		Interaction, β (SE)	*P* value
	Baseline	Follow-up	Baseline	Follow-up		
Perceived Stress Scale–10	25.25 (0.96)	21.38 (0.98)	25.92 (0.98)	18.21 (1.03)	−3.84 (1.37)	*.007* ^b^
Connor-Davidson Resilience Scale–2	4.68 (0.28)	4.81 (0.27)	4.33 (0.27)	5.29 (0.29)	0.83 (0.43)	.06
Generalized Anxiety Disorder–7 scale	11.43 (0.84)	9.68 (0.85)	10.98 (0.85)	6.02 (0.89)	−3.21 (0.97)	*.002*
Patient Health Questionnaire–9	12.15 (1.14)	10.42 (1.15)	11.78 (1.16)	6.93 (1.20)	−3.13 (1.13)	*.007*
General Well-Being Schedule	55.31 (2.53)	59.72 (2.56)	56.65 (2.57)	71.05 (2.67)	9.99 (2.92)	*.001*

^a^Reported means and interaction estimates were derived from linear mixed-effects models that included intervention, time, sex, and the time×intervention interaction.

^b^Italicized *P* values indicate statistical significance.

### Secondary Outcomes

The intervention group showed significant reductions in GAD-7 and PHQ-9 scores and significant increases in CD-RISC2 and GWS scores from baseline to follow-up. Significant interactions were also observed for GAD-7 (β=−3.21, SE 0.97; *P*=.002), PHQ-9 (β=−3.13, SE 1.13; *P*=.007), and GWS (β=9.99, SE 2.92; *P*=.001), but not for CD-RISC2 (β=0.83, SE 0.43; *P*=.06). Detailed results from the sex-adjusted models are shown in Table 2, and results from the unadjusted models are provided in Table S3 in Multimedia Appendix 1.

### Intervention Use

At the end of the intervention period, participants in the intervention group completed an average of 35% of the MR program. When examining the relationship between changes in outcomes and the percentage of modules completed, we did not find a significant interaction between time and the percentage of modules completed. Module completion was also not a significant predictor of changes in outcomes when examined by quartile. The average number of assisted coaching sessions used by participants in the intervention group was 1.3 out of a maximum of 3. Among individuals who attended any coaching sessions, the average number of sessions used was 2.2. No associations were found between the number of coaching sessions and changes in the primary and secondary outcomes. Study participants reported no harmful or unintended consequences.

## Discussion

### Principal Findings

This pilot study aimed to explore the feasibility of mobile VR technology as a potential tool for reducing stress and managing anxiety among young adults. The results showed that after 8 weeks of using a VR-based mindfulness program, the intervention group had significant reductions in stress, anxiety, and depressive symptoms and an increase in QoL.

Our findings are consistent with those of the limited previous studies demonstrating that VR technology, particularly mobile VR, is effective in reducing stress and managing mental health challenges. Gao et al [28] found that the use of mobile VR led to a reduction in stress. Similar findings were reported by McGarry et al [60], who found that mobile VR reduced psychological stress and was perceived as a useful and effective strategy for stress management by young adults aged 18 to 25 years. These 2 studies, however, did not have an active control group. In contrast, a mixed methods study with a control group that examined the short-term pre-post intervention effect on psychological well-being also reported a large decrease in stress, anxiety, and sadness in the intervention group [30]. These findings should be interpreted cautiously, as all 3 studies were limited to sample sizes of <20 participants and brief VR exposures ranging from a single session to a maximum of 14 days. Furthermore, these studies focused on generic VR apps that used scenery for relaxation, whereas our study used an integrated app designed for stress reduction through a CBT-based structured protocol.

Despite the positive findings from our study, participant engagement remains an important area for improvement. The average completion rate of VR sessions was 35% in this 8-week, 76-module intervention. Although modest, this level of completion is consistent with adherence patterns observed in multiweek, self-directed digital mental health programs, where attrition over time is common and full completion rates often fall within similar ranges (reported ranges of 0.5% to 28.6%), particularly in the absence of performance-based incentives [61]. Digital mindfulness and CBT interventions frequently demonstrate a progressive decline in engagement across sequential modules, especially in young adult populations [30,62]. In the present study, the requirement to complete modules sequentially and, for 40 modules, to set up immersive VR hardware may have introduced additional user burden relative to purely smartphone-based apps. Although monthly coaching sessions were incorporated to support adherence, the frequency of support may not have been sufficient to fully counteract typical digital attrition. Future iterations may benefit from increased personalization, adaptive pacing, or gamification strategies to enhance sustained engagement.

Our study did not find a dose-response relationship. In the regression models examining the preintervention and postintervention changes as a function of module completion, there was a trend in which participants in the highest quartile had the greatest reduction in PSS-10, GAD-7, and PHQ-9 scores compared to those in the lowest quartile. However, the differences did not reach statistical significance. Although dose-response relationships have been observed in VR interventions for other conditions such as PTSD and chronic pain, there is a lack of consensus regarding such relationships in VR-based stress management [63,64]. Future studies should explore effective engagement strategies and dose-response effects to strengthen VR-based stress management programs.

The ways in which VR reduces stress and anxiety are likely to be multifaceted. VR creates an immersive environment that can transport users to calm, relaxing settings, and this immersion can help redirect users away from real-world stressors and promote relaxation [65,66]. The VR app used by our participants incorporated guided imagery and relaxation techniques, such as deep breathing exercises and mindfulness practices. In addition, the program also facilitated psychoeducational content grounded in CBT. These techniques help users build coping skills, reduce stress responses, and achieve relaxation [65].

Unlike the significant findings for stress, we did not find a significant improvement in resilience associated with MR. Resilience, defined as the ability to adapt to adversity, is a critical component of mental health and well-being [67]. However, resilience may be influenced by a broader range of factors beyond the scope of our intervention, such as long-term life experiences, social support, and personality traits, which may require more extended or targeted interventions to observe measurable changes. There is a limited body of research examining resilience as an outcome of VR-based stress interventions, but given the potential of resilience to buffer against a poor ability to cope with stress, further research in this area is warranted.

An important observation from our study is the improvement in self-reported QoL following the intervention. QoL is a multifaceted construct of well-being, encompassing physical, psychological, and social dimensions [68]. Stress and anxiety are known to be negatively associated with QoL, contributing to emotional instability, cognitive difficulties, and possibly impaired social interactions [69]. Studies have shown that interventions aimed at reducing stress can improve QoL [27]. This study provides further evidence supporting the relationship between stress and QoL. Unlike generic relaxation VR, which typically consists solely of passive, scenery-based relaxation audiovisual components without targeted educational or skill training content, the intervention used in this study was designed for stress management with structured techniques and guided practices. This targeted approach may be more effective than generic relaxation VR in improving psychological well-being and stress management. Future research should explore how integrating adaptive content and personalized engagement strategies can further optimize VR interventions for sustained improvements in QoL.

As VR technology continues to evolve, its integration into mental health care systems offers more possibilities. Mobile VR solutions, in particular, are advantageous due to their high accessibility and portability, making them widely available and suitable for individuals regardless of location [70]. Furthermore, the increasing prevalence of telehealth provides an opportunity to complement remote therapy with VR-based interventions, offering individuals accessible tools for managing psychological distress at home. The feasibility and cost-effectiveness of integrating VR into existing mental health care services will be an important next step toward maximizing VR’s potential impact.

### Strength and Limitations

Although this study used an RCT design, several limitations remain. First, our study had a small sample size and a relatively short study duration, limiting the ability to assess the intervention’s long-term effects. Participants were not blinded due to the nature of the VR intervention, which may introduce expectancy effects in both the intervention and control groups. Although we observed significant reductions in stress, anxiety, and depressive symptoms over the trial period, the duration of these effects remains uncertain. Additionally, all outcomes were measured using self-reported surveys, which may be subject to reporting bias. The generalizability of our findings is also limited by the self-selected nature of our sample, which consisted primarily of young adults in New York City. The inclusion criteria were necessary to account for confounders but may have inadvertently excluded individuals who would benefit from the intervention in real-world settings. Although many eligible individuals did not proceed to randomization, this attrition occurred between screening and in-person equipment collection, suggesting that logistical requirements may have posed a barrier to study participation. Further research with larger, more diverse samples and extended follow-up periods is necessary to determine the sustained benefits of VR-based stress management interventions over time.

### Conclusions

This study provides preliminary evidence supporting the use of mobile VR technology to reduce stress, anxiety, and depressive symptoms and to improve QoL among young adults. Given that this study had a moderate sample size, a low program completion rate, and a sample that was predominantly female, self-selected, and mostly urban, the generalizability of the study’s results should be approached with caution. Future research should examine the efficacy of this VR app with larger, more diverse samples to assess its long-term mental health impact.

## Data Availability

The dataset used and/or analyzed in this study is available from the corresponding author upon reasonable request.

## Appendix

Multimedia Appendix 1 Supplementary file with additional characteristics and outcome data.

Multimedia Appendix 2 CONSORT (Consolidated Standards of Reporting Trials) checklist.

## Abbreviations

CBT: cognitive behavioral therapy
CD-RISC2: Connor-Davidson Resilience Scale–2
CONSORT: Consolidated Standards of Reporting Trials
CVD: cardiovascular disease
GAD-7: Generalized Anxiety Disorder–7
GWS: General Well-Being Schedule
ITT: intention-to-treat
MR: MindCo Relief
PHQ-9: Patient Health Questionnaire–9
PSS-10: Perceived Stress Scale–10
PTSD: posttraumatic stress disorder
QoL: quality of life
RCT: randomized controlled trial
VR: virtual reality
