# Supplementary material for: A Mobile Virtual Reality–Based Intervention for Stress Reduction Among Young Adults: Pilot Randomized Controlled Trial
Source: JMIR Mhealth Uhealth. 2026 Aug 21;14:e83438. doi: 10.2196/83438 (PMC13545530; doi:10.2196/83438)
Supplement: Supplementary file 2 [file mhealth_v14i1e83438_app1.docx]

Supplementary Table 1. Comparisons of demographics between enrolled vs. non-enrolled but otherwise eligible individuals.

|  | **Eligible but not enrolled** | **Enrolled** |  |
| --- | --- | --- | --- |
|  | **N (%)** | **N (%)** | ***P* value** |
| **Student status** |  |  |  |
| Non-Student | 109 (34.0) | 24 (34.3) | .958 |
| Student | 212 (66.0) | 46 (65.7) |  |
| **Insurance status** |  |  |  |
| Public | 177 (55.1) | 39 (55.7) | .951 |
| Private | 115 (35.8) | 24 (34.3) |  |
| None | 29 (9.0) | 7 (10.0) |  |
| **Sex** |  |  |  |
| Missing | 23 (7.2) | 0 (0.0) |  |
| Female | 97 (30.2) | 18 (25.7) | .267 |
| Male | 201 (62.6) | 52 (74.3) |  |
| **Race** |  |  |  |
| Asian | 97 (30.2) | 25 (35.7) | .747 |
| Black | 66 (20.6) | 10 (14.3) |  |
| Hispanic | 97 (30.2) | 23 (32.9) |  |
| Other | 6 (1.9) | 1 (1.4) |  |
| White | 55 (17.1) | 11 (15.7) |  |
|  |  |  |  |
|  |  |  |  |
|  | **Mean (SD)** | **Mean SD** | ***P* value** |
| Age | 21.96 (3.12) | 23.36 (3.41) | <.001 |

Note: *P* value generated for sex excluded the individuals with missing values.

Supplementary Table 2. Comparison between individuals lost to follow-up with those with follow-up data.

|  | **Lost to follow-up** | **Follow-up provided** |  |
| --- | --- | --- | --- |
|  | **N (%)** | **N (%)** | ***P* value** |
| **Intervention status** |  |  |  |
| Control | 2 (33) | 34 (53) | .42 |
| Intervention | 4 (67) | 30 (47) |  |
| **Student status** |  |  |  |
| Non-Student | 1 (17) | 23 (36) | .34 |
| Student | 5 (83) | 41 (64) |  |
| **Insurance status** |  |  |  |
| Public | 2 (33) | 37 (58) | .51 |
| Private | 3 (50) | 21 (33) |  |
| None | 1 (17) | 6 (9) |  |
| **Sex** |  |  |  |
| Female | 2 (33) | 16 (25) | .64 |
| Male | 4 (67) | 48 (75) |  |
| **Race** |  |  |  |
| Asian | 2 (33) | 22 (34) | .57 |
| Black | 2 (33) | 7 (11) |  |
| Hispanic | 1 (17) | 21 (33) |  |
| Other | 0 (0) | 4 (6) |  |
| White | 1 (17) | 10 (16) |  |
|  |  |  |  |
|  |  |  |  |
|  | **Mean (SD)** | **Mean SD** | ***P* value** |
| Age | 23.33 (3.44) | 23.33 (3.43 | .99 |

Note: Fisher’s exact test was used given cell counts less than 5.

Supplementary Table 3. Estimates from the unadjusted model.

| **Unadjusted model** | | | | | | |
| --- | --- | --- | --- | --- | --- | --- |
|  | **Intervention** | | **Time (Beta ± SE)** | | **Interaction** | |
| **Outcomes** | **Beta ± SE** | ***P* value** | **Beta ± SE** | ***P* value** | **Beta ± SE** | ***P* value** |
| PSS-10 Total | 0.65 (1.30) | .616 | -3.88 (0.95) | <.001 | -3.81 (1.38) | **.007** |
| CD-RISC Total | -0.71 (0.74) | .344 | 0.27 (0.61) | .662 | 1.66 (0.86) | .058 |
| GAD-7 Total | -0.46 (1.12) | .680 | -1.75 (0.67) | .011 | -3.20 (0.97) | **.002** |
| PHQ-9 Total | -0.37 (1.52) | .807 | -1.73 (0.78) | .030 | -3.13 (1.13) | **.007** |
| GWS Total | 1.35 (3.37) | .689 | 4.41 (2.01) | .032 | 9.98 (2.92) | **.001** |
|  |  |  |  |  |  |  |

Note: The unadjusted model was derived from a linear mixed-effects model that included intervention, time, and the intervention × time interaction. Bolded *P* values indicate statistical significance.

Abbreviations: PSS-10 – Perceived Stress Scale-10, CD-RISC - Connor-Davidson Resilience Scale, GAD7 – General Anxiety Disorder – 7, PHQ-9 – Patient Health Questionnaire-9 , GWS – General Wellbeing Schedule
